# Understanding the Risks of Diffusion of Cyanobacteria Toxins in Rivers, Lakes, and Potable Water

**DOI:** 10.3390/toxins15090582

**Published:** 2023-09-20

**Authors:** Amin Mahmood Thawabteh, Hani A Naseef, Donia Karaman, Sabino A. Bufo, Laura Scrano, Rafik Karaman

**Affiliations:** 1 Faculty of Pharmacy, Nursing and Health Professions, Birzeit University, Ramallah 00972, Palestine; athawabtah@birzeit.edu (A.M.T.); hshtaya@birzeit.edu (H.A.N.); 2 General Safety Section, General Services Department, Birzeit University, Bir Zeit 71939, Palestine; 3 Faculty of Pharmacy, Al-Quds University, Jerusalem 20002, Palestine; kdonia65@yahoo.com; 4 Department of Sciences, University of Basilicata, Via dell’Ateneo Lucano 10, 85100 Potenza, Italy; sabino.bufo@unibas.it; 5 Department of Geography, Environmental Management and Energy Studies, University of Johannesburg, Auckland Park Kingsway Campus, Johannesburg 2092, South Africa; 6 Department of European and Mediterranean Cultures, University of Basilicata, Via Lanera 20, 75100 Matera, Italy; laura.scrano@unibas.it

**Keywords:** cyanobacteria blooms, cyanotoxins, *Microcystis*, *Anabaena*, cylindrospermopsin, satellite imagery, biocontrol

## Abstract

Blue-green algae, or cyanobacteria, may be prevalent in our rivers and tap water. These minuscule bacteria can grow swiftly and form blooms in warm, nutrient-rich water. Toxins produced by cyanobacteria can pollute rivers and streams and harm the liver and nervous system in humans. This review highlights the properties of 25 toxin types produced by 12 different cyanobacteria genera. The review also covered strategies for reducing and controlling cyanobacteria issues. These include using physical or chemical treatments, cutting back on fertilizer input, algal lawn scrubbers, and antagonistic microorganisms for biocontrol. Micro-, nano- and ultrafiltration techniques could be used for the removal of internal and extracellular cyanotoxins, in addition to powdered or granular activated carbon, ozonation, sedimentation, ultraviolet radiation, potassium permanganate, free chlorine, and pre-treatment oxidation techniques. The efficiency of treatment techniques for removing intracellular and extracellular cyanotoxins is also demonstrated. These approaches aim to lessen the risks of cyanobacterial blooms and associated toxins. Effective management of cyanobacteria in water systems depends on early detection and quick action. Cyanobacteria cells and their toxins can be detected using microscopy, molecular methods, chromatography, and spectroscopy. Understanding the causes of blooms and the many ways for their detection and elimination will help the management of this crucial environmental issue.

## 1. Introduction

**Figure 1:**
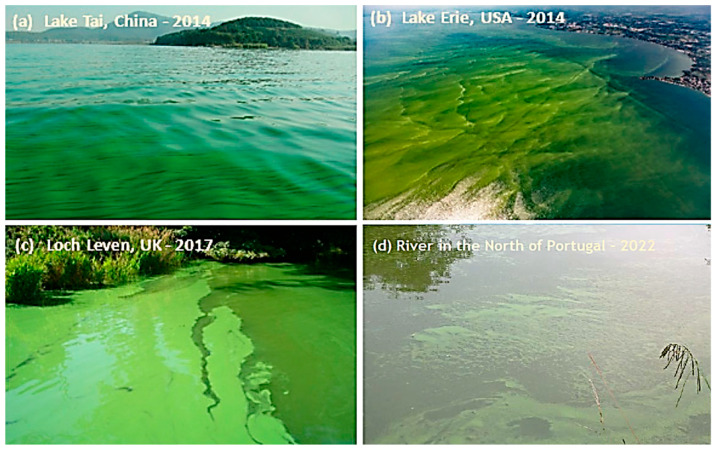
Cyanobacterial growth and dissemination on lake (**a**–**c**) and river (**d**) surfaces [3,8]. Reproduced with permission from Tao Lyu, Lirong Song, Qiuwen Chen, and Gang Pan, Water Journal; published by MDPI, 2020. Reproduced with permission from Moreira, C.; Vasconcelos, V.; Antunes, A., Earth Journal; published by MDPI, 2022.

On occasion, drinking tap water results in an earthy flavor that is detectable right away. In addition to colored scum that can develop on river surfaces, foam can occasionally form on lake or rive’ surfaces. Cyanobacteria are depicted in Figure 1 [1,2,3,4] as the colorful scum and the earthy taste of tap water. This unpleasant, old-growth organism contaminates many of our freshwater supplies, which threatens human health. While cyanobacteria are naturally present in our rivers and water supply, green growth and environmental modifications brought on by human activity might hasten their spread [2,3,4,5,6,7]. Our rivers and tap water may include cyanobacteria. These tiny bacteria can swiftly produce blooms in warm, nutrient-rich water, endangering humans and aquatic life. Toxins produced by cyanobacteria include *Microcystins*, which are well-known for their toxicity and capacity to harm plants. These poisons contaminate rivers and streams and threaten our drinking water supply since they can sneak through standard water treatment systems unnoticed [6,7,8].

Since these bacteria thrive in warmer climates, they pose a severe risk when river levels rise in the summer. To make matters worse, getting rid of cyanobacteria from nearby bodies of water can be challenging once a bloom has occurred. Because of this, we must comprehend the dangers of cyanobacteria and how to stop their spread in our drinking water [2,7,8,9].

When there is an abundance of warm, stagnant water and high amounts of phosphorus and nitrogen from sources like agricultural runoff or untreated sewage, cyanobacteria blooms commonly take place. Additionally, elements like global warming and the frequency of extreme weather events may make these blooms more likely [8,9,10].

The summer months experience mild temperatures, when these blossoms most frequently appear. They can also happen throughout extended periods of low precipitation and dryness. Due to long-term excess nutrient loading in a specific body of water, heavy rains may occasionally result in cyanobacterial blooms [11].

It is essential to remember that blooms can occur at any moment, so it is best to exercise caution when swimming in water bodies susceptible to blooming. Even if tests show that a river or lake’s cyanobacterial toxin levels are low, if a bloom happens when a person is swimming there, they may still be at risk [10,11,12]. In more detail, we will examine the topic in this review, learning where cyanobacteria originate, how they spread, and what dangers come with this form of toxicity diffusion in our waters. We will also discuss some measures implemented to reduce these hazards and maintain the safety of our tap water.

## 2. Common Genera of Cyanobacteria and Their Characteristics

In practically every freshwater ecosystem, cyanobacteria can be found alone or in combination with other organisms. In addition, there are numerous distinct cyanobacteria genera, each with unique properties. The common cyanobacteria genera discovered in freshwater systems are *Synechococcus*, *Anabaena*, *Rivularia*, *Gloeotrichia*, *Oscillatoria*, *Cylindrospermopsis*, *Aphanizomenon*, *Planktothrix*, *Scytonema*, *Tolypothrix*, *Merismopedia*, *and Microcystis* [13,14,15,16]. 

### 2.1. Microcystis: Colonial, Spherical Cells, Toxin Producer, Toxic, and Bloom-Forming

One of the most prevalent genera of cyanobacteria found in freshwater environments is *Microcystis*, a genus of colonial, spherical cells. *Microcystis* can produce toxins; they are poisonous and can cause blooms. Their colonies can be any shade of green, from deep olive to vivid blue-green. *Microcystis* colonies expand swiftly and have a milliliter cell density of over 1 million [14,15,16]. They prefer to form mats on the water’s surface along with other species like *Anabaena*, *Oscillatoria*, or *Aphanizomenon* and are photosynthetic organisms [17,18,19].

Moving water like rivers or streams and standing water like ponds, lakes, or reservoirs can harbor *Microcystis*. Because of the lower levels of oxygen and warmer temperatures throughout the summer, blooms are more likely to happen. Due to decreased oxygen levels and the development of chemicals that can harm humans and plant life, the blooms may result in drop-down water quality [20,21].

### 2.2. Anabaena: Filamentous, Heterocysts for Nitrogen Fixation and Diazotrophic

Anabaena inhabits environments in freshwater. These cyanobacteria are particularly important for their capacity to fix nitrogen since they include heterocyst cells capable of doing so. *Anabaena* also has akinetes cells, which act as protective spores and can withstand harsh environmental conditions [22,23]. 

The filaments that make up an *Anabaena* comprise individual trichome cells, which are one cell thick. Each trichome contains photosynthetic cells with a thick cell wall and an external peptidoglycan coating. The nucleoids, thylakoids, and carboxysomes of the cells are home to the enzymes involved in photosynthesis. Along the length of the trichome, heterocysts act as the central locations for nitrogen fixation [23,24,25]. *Anabaena* has undergone extensive research due to its ability to produce diazotrophic endospores that can be used to study other cyanobacterial species further. It is commonly used in aquatic systems to increase their nutritional content because of its ability to fix atmospheric nitrogen [25,26]. By doing so, it can supply other species with fixed nitrogen in settings where it is scarce or absent.

### 2.3. Oscillatoria: Filamentous, Motile, No Heterocysts

*Oscillatoria* is a widespread genus of freshwater cyanobacteria, the bacteria that cause bodies of water to get murkier and greener. It has a slimy, thin filamentous structure. Inhabiting shallow and deep regions of nutrient-rich water, such as seas, rivers, and lakes, *Oscillatoria* prefers to coil in an oscillatory motion [27,28].

It belongs to the cyanobacteria family and is not heterocytic, meaning it does not have heterocysts, a specific cell that fixes nitrogen in plants’ roots. *Oscillatoria* is frequently grouped with the Chroococcales family regarding their growth and physiological circumstances [29].

Under some circumstances, these mobile bacteria can create floating mats of colonies on the water’s surface. *Oscillatoria* can withstand dry seasons’ droughts thanks to the viscous slime that coats its cell walls. Furthermore, by utilizing nitrates to generate energy, it can survive in low light or even complete darkness because of its unique photosynthesis style [28,29,30].

### 2.4. Cylindrospermopsis: Filamentous, Motile, Toxin Producer

A filamentous and mobile genus of cyanobacteria called *Cylindrospermopsis* makes toxins. Freshwater systems like lakes and rivers and artificial reservoirs like ponds and canals frequently contain it. In temperate areas, this genus displays sporadic flowers in the summer [31].

*Cylindrospermopsis* spreads swiftly and takes over a system due to its motile habit, which makes it tough to eradicate. Despite being filamentous creatures, they can develop both deep blooms that can extend several meters below the surface of the water and enormous mats close to the water’s surface [32,33].

In addition to hepatotoxins (cyanotoxins), neurotoxins (ciguatoxins), and components of bacterial cell walls (lipopolysaccharides or LPS), *Cylindrospermopsis* species also produce toxins. Scientists must watch for these species in freshwater systems because these cyanotoxins are very hazardous to people and animals, even at low concentrations [32,33,34].

### 2.5. Aphanizomenon: Filamentous, Motile, Nitrogen Fixer

The genus Aphanizomenon of motile, free-floating filamentous cyanobacteria is widely found in freshwater bodies of water. They are known as nitrogen fixers because they can convert atmospheric nitrogen into a form that plants can utilize. As a result, they contribute nutrients to the food chain and maintain high water quality, making them vital to aquatic environments [35,36]. Certain Aphanizomenon species can harm humans and other animals when present in more significant proportions than usual. To further comprehend the potential risks these cyanobacteria pose, it is essential to frequently sample water and evaluate the levels of cyanobacterial metabolites [37]. Aphanizomenon is usually found in blossoms; therefore, that should also be considered. This indicates that an increase in their population may be caused by their quick growth, spurred by an abundance of nutrients or higher temperatures. It can help to prevent possible damage from increased toxin production or other negative impacts on aquatic plants and fauna by routinely checking for bloom indications [38,39].

### 2.6. Planktothrix: Filamentous, Motile, No Heterocysts

Numerous distinct species of *cyanobacteria* that have evolved to flourish in freshwater ecosystems comprise the *Planktothrix* genus. *Planktothrix agardhii*, *P. rubescens*, and *P. limnetica* are the most prevalent species. Due to their preference for nutrient-rich waters, these organisms are frequently found in eutrophic lakes with high levels of phosphate and nitrogen [40,41,42].

Phytotoxicity diffusion is the process of producing toxic exudates on the cell walls of *Planktothrix* species due to dense blooms that decompose. Due to the pigments (such as carotenoids) they produce, *Planktothrix* blooms can also cause water to become discolored [43]. In addition, *Planktothrix* is renowned for its capacity to multiply quickly and, under the right circumstances, to create dense aggregates or mats at the water’s surface. Optimal temperatures for *Planktothrix* development and survival in freshwater systems are between 15 and 25 °C and photosynthetically active radiation (PAR) [43,44].

### 2.7. Synechococcus: Unicellular and Colonial

The most prevalent genus of cyanobacteria is *Synechococcus*. It can be single-celled or colonial, consisting of one or more cells that can cluster and form filaments or colonies containing single or multiple cells. These colonies typically have a spherical form, a single gas exchange aperture, and a polysaccharide wall [45,46].

*Synechococcus* is renowned for its capacity to endure harsh conditions, including acidic and hypersaline waters. Due to its tolerance for high salt concentrations, it is also a significant species in coral reefs and soils, both aquatic environments. Its capacity for photosynthesis has also made it a vital component of the oxygen cycle in aquatic settings [47,48,49].

### 2.8. Rivularia: The Bubble-like Colonies 

*Rivularia* create colonies that resemble bubbles, with each cell living inside a separate, protective “home”. Several characteristics set *Rivularia* apart from other cyanobacterial taxa. First, unlike most other cyanobacteria, *Rivularia* colonies are relatively solid in the water. The individual cells each have a distinct shape, with flat bottoms and rounded tops [50,51,52].

Additionally, depending on their environment, *Rivularia* colonies can appear very varied. When exposed to large amounts of light or oxygen, the colonies turn a deeper shade of green or even blue-black. However, colonies deprived of oxygen and light become paler and more transparent [53,54].

### 2.9. Gloeotrichia: Filamentous Cyanobacteria with Distinct Branching

The *Gloeotrichia* genus is distinguished by its distinctive branching, which gives its members the appearance of a starburst or a dandelion gone berserk. Due to their characteristic morphology, *Gloeotrichia* members are frequently observed in freshwater or brackish water habitats. They are anoxygenic phototrophs, producing sulfur rather than needing oxygen to use the light energy from photosynthesis [55,56].

These creatures’ internal sheaths have a spiral arrangement of cells that facilitates their movement through their environment, improving nutrition intake and mobility throughout your aquascape. The cell’s structure and surface area influence its interaction with its surroundings [56,57,58].

Most organisms have a variety of photosynthetic pigments in their photosynthetic system, allowing them to utilize light from locations where it is most abundant efficiently. Additionally, they have a variety of flagella to assist them in moving around in water deficient in nutrients or containing a lot of dissolved materials, such as salts and metals [58,59].

### 2.10. Scytonema: Irregularly Branched Filaments

Cyanobacteria belonging to the genus *Scytonema* have filaments that are erratically branched. When growing in colonies, cells can form cords, tufts, and even mats and are typically oriented spirally. The second name of this particular cyanobacterium species refers to the color of its distinctive brilliant reddish-brown spores. It can be found on mosses and lichens and often grows on damp surfaces [60,61].

*Scytonema* stands out from other cyanobacteria in several ways. It does not require light for photosynthesis, enabling it to flourish in dark places like caves or deep-sea cracks. A bud forms from the side of the bacteria cell and splits off to create a new organism, which is how they mainly reproduce [61,62]. 

They can also continue for extended periods without water because they enter a latent stage when they do not require oxygen. On the other hand, several species have unique pigments that enable them to absorb various light wavelengths, allowing them to adapt to multiple habitats [61,62,63].

### 2.11. Tolypothrix: Pseudoparenchymatous Filaments

Because of its pseudo-parenchymatous filaments, *Tolypothrix* can be identified. As a filamentous cyanobacterium that resembles a thread, *Tolypothrix* can join with other *Tolypothrix* cells to form lengthy chains of cells. This genus is extensively distributed worldwide and can be found in fresh and saltwater settings [64]. 

Typically comprised of four to six cells, *Tolypothrix* cells are protected from external challenges like UV radiation and drying out by a rigid coating consisting of glycoproteins and polysaccharides. The glycoprotein sheath keeps Their cell walls together, making it easier for them to build long chains [64,65,66].

Contrary to other cyanobacterial genera, such as *rivularia* or *Gloeotrichia*, *Tolypothrix* filaments rely on robust glycoprotein sheaths to make these connections rather than a visible sheath or stalk-like structure. This distinctive structure provides them a distinct edge when adapting to various settings, which explains why they are such a widespread genus globally [65,67]. 

### 2.12. Merismopedia: Cubicpacket-Shaped Cells

*Merismopedia*, a common genus of cyanobacteria, has a distinctive cubic packet-shaped cell and can split up to four times before finally dispersing. *Merismopedia* cells typically come in two shapes: flattened and cubed, ranging in size from 2 to 6 m [68,69]. When these cells cluster together in their surroundings, their morphology enables them to create a slimy, jelly like substance. This characteristic slimy mass typically comprises numerous layers with various types of *Merismopedia* cells [70].

Although it can also be found in wet soil and other moist habitats, *Merismopedia* is primarily found in oceans, seas, and other aquatic environments. Additionally, *Merismopedia* is frequently linked to cyanobacterial mats because of its ability to adapt to different light, temperature, and salt concentrations [71,72].

*Merismopedia* needs specific minerals like potassium and nitrogen to thrive appropriately and does it best in direct sunshine. It can grow rather quickly under ideal circumstances, such as temperatures between 25 and 35 °C and pH levels between 6 and 8.1. It is crucial to understand where *Merismopedia* is typically located and how to recognize it because this tiny but mighty genus of cyanobacteria can create toxins that can be dangerous to people if swallowed or even inhaled [71,72,73].

## 3. Cyanobacteria Diffusion and Spreads in Water Bodies

When cyanobacteria, single-celled organisms, disperse in water bodies, a process known as cyanobacteria diffusion and spread takes place. This may lead to higher concentrations of blue-green algae, or cyanobacteria, in certain conditions. It is critical to comprehend the elements that encourage bloom production and dissemination to understand the spread of this group of organisms [74,75,76].

### 3.1. Natural Factors Promoting Cyanobacteria Bloom Formation and Diffusion

**Figure 2:**
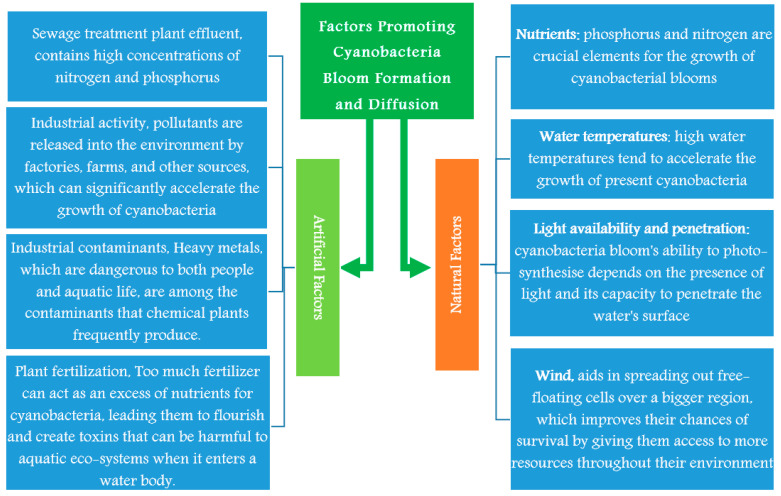
Factors promoting cyanobacteria bloom formation and diffusion.

The availability of nutrients greatly influences blooms, and both phosphorus and nitrogen are crucial elements for the growth of cyanobacterial blooms. As a result, it is expected that some bloom may develop in water bodies where these nutrients are present in high concentrations [76,77,78]. Other factors as water temperatures, light availability and penetration will be discussed in this section and Figure 2.

First of all, phosphorus promotes cyanobacterial development and the production of toxic toxins. This is so it can function appropriately in cyanobacterial photosynthesis and cellular metabolism. Additionally, an overabundance of phosphorus can cause populations of cyanobacteria to increase quickly, resulting in a bloom [79]. On the other hand, because it is both a source of energy and a crucial element for the synthesis of cyanobacteria biomolecules, nitrogen also significantly impacts bloom development. As a result, too much nitrogen can alter the chemistry of the water, which can lead to a bloom [79,80].

Conversely, high water temperatures significantly aid cyanobacteria bloom development and dissemination. A body of water stratifies into two or more strata (thermoclines) according to its density as the temperature rises. This stratification prevents vertical mixing, which would otherwise separate nutrients near the surface from those in deeper waters, giving the cyanobacteria a surplus of food resources [81]. Additionally, a decline in vertical mixing may cause the near-surface waters to stagnate. Due to their tolerance to low oxygen levels, cyanobacteria can flourish in oxygen-depleted systems, creating the perfect setting for their growth. Additionally, high water temperatures tend to accelerate the growth of present cyanobacteria, which promotes the creation and diffusion of blooms [81,82,83].

Light availability and penetration are the third elements favoring the creation and diffusion of cyanobacterial blooms. The cyanobacteria bloom’s ability to photosynthesize depends on the presence of light and its capacity to penetrate the water’s surface. There are two types of light availability; depending on the atmosphere, it may be direct or indirect. While indirect light is sunlight reflected off an object or surface before entering the water, natural light enters the water directly but may be constrained by shadowing from other creatures. This could lead to uneven lighting, which would impact cyanobacteria blooms’ photosynthesis rate differently than direct light [83,84,85,86].

It is necessary to consider light penetration via water in addition to direct and indirect light. This depends on variables such as those that alter water clarity due to dissolved organic matter and the presence of other species suspended in the water column, which may limit the depth to which sunlight can penetrate. Knowing how light penetration and availability affect cyanobacteria blooms helps us understand where, when, and why these blooms occur as well as how to prevent them from occurring in undesirable locations [87,88].

The growth and diffusion of cyanobacteria blooms are significantly aided by water mixing and movement. One of the leading promoters or inhibitors of blooms is hydrodynamics, the study of the movement of fluids, which includes ocean currents, water flow, and wave action. Geographical location, seasonality, and a variety of environmental conditions, like as temperature gradients, all have a role in this. For example, a large-scale temperature gradient in the water might boost mixing, which is suitable for algae development—because of the blending, nutrients from the bottom rise to the surface, giving the algae more food [88,89,90]. 

Another significant aspect in the production and dispersion of cyanobacteria blooms is wind because it helps to stir up the water column, which again aids in bringing vital nutrients up from the bottom for algae growth. Additionally, it aids in spreading out free-floating cells over a more extensive region, which improves their chances of survival by giving them access to more resources throughout their environment [91,92].

These are all strongly connected to human activity, including climate change and eutrophication from excessive fertilizer runoff. It is well known that the presence of these elements alters the behavior of bacteria: they become less mobile, and their metabolic activity rises, assisting them in settling on substrates and remaining there more quickly. This causes cyanobacteria to spread throughout a river or even into drinkable water sources.

### 3.2. Artificial Factors Promoting Growth of Cyanobacteria Bloom Formation

The main artificial factors promoting the growth of cyanobacteria bloom formation are sewage treatment plant effluent, industrial activity, industrial contaminants, and plant fertilization; these sources will be discussed in this section and Figure 2.

One of the most critical sources of cyanobacterial toxicity in aquatic bodies is sewage treatment plant (STP) effluent. This is because STP effluent typically contains high concentrations of nitrogen and phosphorus released into water bodies, creating the perfect conditions for cyanobacteria to flourish. Additionally, these nutrients may build up in the water, causing eutrophication. As a result, cyanobacteria and other microbes proliferate quickly, causing blooms that may be hazardous to aquatic life. By disrupting oxygen levels and elevating pH levels, the chemicals emitted by these blooms can harm both plants and animals [93,94,95,96,97].

Wastewater treatment facilities must be appropriately maintained to ensure adequate nutrient removal from the effluent before it enters the receiving waterbody. This will help to limit the effects of STP effluents on water bodies. Additionally, to minimize excessive algal growth and lessen its impact on aquatic life, effective management measures should be implemented when dealing with excessive nutrients from agricultural runoff or urban areas [98,99,100,101,102].

Industrial activity is one of the main causes of cyanobacterial toxicity. Pollutants are released into the environment by factories, farms, and other sources, which can significantly accelerate the growth of cyanobacteria, or blue-green algae [103,104,105]. Nutrient runoff is the main factor driving the proliferation of cyanobacteria. Rainfall typically causes neighboring water bodies to rapidly expand in population by introducing nutrients like nitrogen and phosphorus [105,106,107,108,109].

Other industrial contaminants also aid the development of phytotoxic cyanobacteria. Heavy metals, dangerous to both people and aquatic life, are among the pollutants that chemical plants frequently produce. Additionally, agricultural practices can lead to a rise in contaminants like pesticides and fertilizers, which can lower the quality of water bodies by raising the likelihood of cyanobacterial growth [110,111,112,113,114,115,116].

Plant fertilization can result in cyanobacterial growth. Too much fertilizer can produce an excess of nutrients for cyanobacteria, leading them to flourish and create toxins that can harm aquatic ecosystems when they enter a water body. Therefore, it is crucial to take care while applying fertilizer close to their local water bodies [117,118,119]. In addition to causing cyanobacterial toxicity, excessive fertilizer use also causes eutrophication, in which algae density and biomass exceed normal levels due to an abundance of nutrients, depleting the water’s oxygen content and upsetting the local ecosystem [120,121,122,123].

## 4. The Common Toxins Produced from Cyanobacterial Blooms 

Hazardous toxins, also called cyanotoxins, are present in cyanobacterial blooms. These cyanotoxins have been linked to several health issues, including cancer, liver damage, neurological problems, rashes, and skin irritation. Hepatotoxins, neurotoxins, dermatoxins, cytotoxins, and endotoxins are the most typical cyanotoxins produced by water blooms [124,125,126,127,128].

Hepatotoxins and neurotoxins are the two most prevalent cyanobacterial toxins. Hepatotoxins are harmful substances that harm the liver and can result in nausea, vomiting, and in severe cases, jaundice. When the neurological system is targeted by neurotoxins, symptoms such as headaches, nausea, muscle weakness, and even paralysis may result from exposure to excessive dosages. In addition, there are reproductive poisons that harm both men’s and women’s reproductive systems. If pregnant women are exposed to dangerous levels of cyanobacteria, this can lead to infertility or even miscarriage [126,127].

Hepatotoxic conditions are brought on by cyanotoxins, among which microcystin is one of the most often produced by cyanobacteria. Hepatotoxic chemicals build up in the water during dangerous cyanobacterial blooms, where they can be consumed through contaminated drinking water or inhaled. When a toxin enters the bloodstream and builds up in the liver, it damages the liver cells by rupturing their membranes [129,130,131].

Abdominal pain, nausea, and vomiting, as well as weakness, exhaustion, jaundice, dark urine, and abnormal liver enzymes, are indications of this disruption. Acute liver failure and possibly death can result from microcystin poisoning in severe situations [132,133,134]. 

When consuming food from polluted water, neurotoxic shellfish poisoning (NSP), which is brought on by the cyanotoxin anatoxin-A, is a serious cause for concern. Research suggests that it attaches to nicotinic acetylcholine receptors and interacts with our nervous system. This binding causes motor neurons to become paralyzed, which can cause problems with coordination, weakness, and in severe cases, even death [135,136].

However, it may take up to 12 h for symptoms to appear. They could include nausea, vomiting, headaches, and dizziness. When ingested in high doses, anatoxins can cause respiratory failure and muscle paralysis, which, if not treated right away, can soon result in death [136,137]. 

The common toxins created by cyanobacterial blooms is described in this section, and the analysis, methodologies, and mitigation techniques of cyanobacteria and cyanotoxins in water are discussed in Section 5.

### 4.1. Microcystin

**Figure 3:**
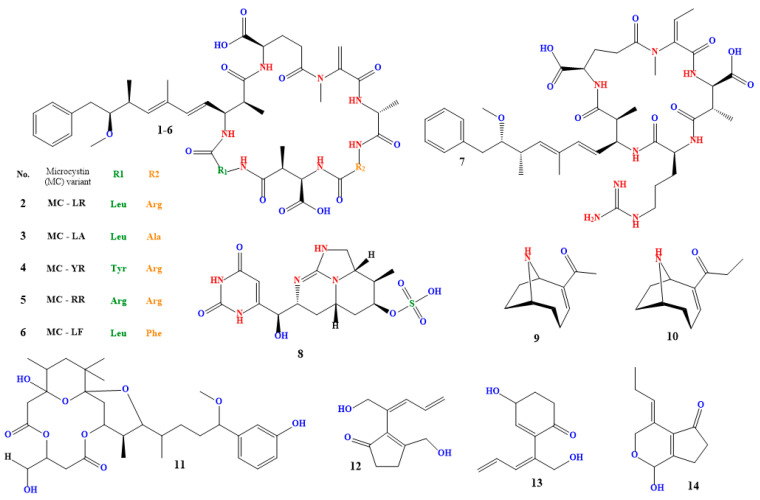
Chemical structures of microcystin (**1**), microcystin-LR (**2**), microcystin-LA (**3**), microcystin-YR (**4**), microcystin-RR (**5**), microcystin-LF (**6**), Nodularin (**7**), Cylindrospermopsin (**8**), Anatoxin-a (**9**), Homoanatoxin (**10**), Oscillatoxin A (**11**), and Nakienones A–C (**12**–**14**).

A class of cyclic hepatotoxins known as microcystins (compound **1** in Figure 3) are produced by many cyanobacterial species, including *Microcystis aeruginosa*, *Anabaena* spp., and *Planktothrix* spp. It is one of the cyanobacterial blooms’ most often made cyanotoxins [129,130,131].

Small peptides known as microcystins can be classified into various structural groups according to the amino acid make-up of their molecules [132,133]. Microcystin-LR (compound **2** in Figure 2), which has a leucine (L) and an arginine (R) residue in its structure, is the most extensively researched and toxic microcystin. Among others [134,135,136,137,138], other microcystin variants include microcystin-LA, microcystin-YR, microcystin-RR, and microcystin-LF (compounds **3**–**6** in Figure 3).

Microcystins are toxic to liver cells and can cause serious liver damage or even liver cancer in both humans and animals. They inhibit protein phosphatases, which are responsible for removing phosphate groups from proteins [139]. Because of the accumulation of phosphorylated proteins in liver cells, normal cellular function is disrupted, which is bad for the liver [140]. The kidney, reproductive system, and liver are all adversely affected by microcystins [141,142,143].

Ingestion of tainted water, skin contact, or inhalation of aerosolized water droplets containing the toxin are all ways people can be exposed to microcystins [141,142]. Although various nations may have their standards and laws regarding cyanotoxins in drinking water, the World Health Organization (WHO) has set a provisional recommendation value for microcystin-LR in drinking water of 1 g/L [143,144].

### 4.2. Nodularin

*Nodularia spumigena*, *Nodularia harveyana*, and *Nodularia moravica* are three species of cyanobacteria that generate nodularin (compound **7** in Figure 3), a cyclic hepatotoxin [145,146,147]. It exhibits the same toxicity mechanism as microcystin and is structurally identical to it. Nodularin inhibits protein phosphatases, which causes phosphorylated proteins to accumulate in liver cells and harm the liver. Nodularin is known to affect other organs, including the kidneys, and has been associated with the development of tumors in test animals [148,149,150].

Nodularin exposure can happen when a person consumes tainted water, comes into contact with it while touching their skin, or breathes in water droplets that have been aerosolized and contain the toxin [151,152,153,154]. Nodularin in drinking water has a provisional guideline value of 0.2 µg/L, according to the World Health Organization (WHO) [155,156,157,158].

### 4.3. Cylindrospermopsin

The cyanobacteria *Cylindrospermopsis raciborskii*, which is currently known as *Raphidiopsis raciborskii* and *Aphanizomenon ovalisporum* generate the toxin known as cylindrospermopsin (compound **8** in Figure 3). It is a tricyclic alkaloid that can be hazardous to humans and animals in acute and chronic phases [159,160,161,162]. Strong liver toxins, such as cylindrospermopsin, can cause cirrhosis and liver necrosis in people and experimental animals. In severe cases, it can also harm the kidneys, leading to renal failure. Cylindrospermopsin has also been shown to cause oxidative stress and DNA damage in cells [163,164].

When contaminated water is drunk, comes into contact with the skin, or is breathed as aerosolized water droplets, cyanospermopsin exposure can occur [165,166]. The World Health Organization (WHO) has set a preliminary recommended threshold for cylindrospermopsin in drinking water at 1 µg/L [167,168,169].

### 4.4. Anatoxin-a

*Anabaena* spp., *Aphanizomenon* spp., and *Cylindrospermum* spp. are cyanobacteria species that generate the powerful neurotoxin anatoxin-a (compound **9** in Figure 3). A secondary, bicyclic amine alkaloid with acute neurotoxicity, anatoxin-a is also referred to as Very Fast Death Factor. It was initially found in Canada in the early 1960s, and it was isolated in 1972. The potent nicotinic acetylcholine receptor agonist anatoxin-A irreversibly binds to these receptors in the central and peripheral nervous systems. As a result of the receptors being overstimulated, the release of the neurotransmitter acetylcholine, which causes muscle contractions, occurs. At high doses, anatoxin-A can cause respiratory collapse and death [170,171].

Ingestion of tainted water, skin contact, or inhalation of water droplets that have been aerosolized can all result in anatoxin-a exposure. Animal deaths and illnesses brought on by cyanobacterial blooms have frequently been attributed to anatoxin-a. The World Health Organization (WHO) has established a provisional guideline value for anatoxin-a in drinking water of 0.2 µg/L [172,173].

### 4.5. Homoanatoxin

*Anabaena circinalis* and *Anabaena lemmermannii* are cyanobacteria species that generate the toxin known as homoanatoxin (compound **10** in Figure 3). It is a cyclic alkaloid and a member of the anatoxins toxin subclass [174,175,176,177].

Homoanatoxin is a powerful neurotoxin that functions as a cholinergic agonist, much like anatoxin-a. In the central and peripheral neurological systems, it binds to nicotinic acetylcholine receptors, overstimulating the receptors and causing the release of acetylcholine, a neurotransmitter that causes muscle contractions. At high doses, homo-anatoxin can cause respiratory collapse and death [178].

The ingestion of tainted water, skin contact, or inhalation of water droplets that have been aerosolized can all lead to homoanatoxin exposure. Numerous cases of animal fatalities and human illness linked to cyanobacterial blooms have been attributed to homoanatoxin [179,180].

### 4.6. Oscillatoxin A

Several cyanobacterial species, such as *Oscillatoria* spp. and *Phormidium* spp., produce the toxin known as oscillatoxin A (compound **11** in Figure 3). It has strong hepato- and neurotoxic properties [181,182]. Oscillatoxin A is a cyclic peptide toxin that shares structural similarities with microcystins. Protein phosphatases 1 and 2A, crucial enzymes involved in various cellular functions like cell division, metabolism, and death, are strongly inhibited by it. Cell damage, particularly in the liver and neurological system, can result from this inhibition [183].

Risks for exposure include drinking polluted water and consuming fish or shellfish that have collected oscillatoxin A. In some instances of animal deaths and human illness, liver damage and neurological symptoms, including paralysis, have been connected to it [182,183,184].

### 4.7. Nakienones A–C

Toxins known as nikienones A–C (compounds **12**–**14** in Figure 3) are made by specific cyanobacteria, such as *Nostoc* and *Anabaena*. They are referred to as lipopeptides and are cytotoxic and antifungal. While nakienone C comprises three separate non-cyclic polypeptides, nakienones A and B are cyclic heptapeptides [185,186].

Toxins from nikienones A–C can cause the body harm, such as digestive problems, skin rashes, liver damage, infertility, and even death. Furthermore, it has been established that each of the three may have medical applications. Nakienone A is an anti-inflammatory and an antifungal, whilst nakienone B and C may potentially inhibit the proliferation of cancer cells. In some cell cultures, nikienone B has been found to have an anti-prostate cancer effect [185,186,187].

It is critical to remember that, like all cyanotoxins, these toxins can be deadly when consumed or inhaled in high doses, even though they may have qualities that make them useful for some medical therapies [188].

### 4.8. Aphantoxin

Aphantoxin made by *Anabaena flosaquae* and *Aphanizomenon* spp. It is a neurotoxin and a member of the PSPs (paralytic shellfish poisons) family. Aphantoxins work by blocking the neurotransmitters that regulate the relaxation and contraction of human muscle [189,190]. This results in severe muscle cramps or paralysis along with a variety of additional systemic symptoms like vomiting or altered mental states [191].

Consuming tainted water or shellfish that have collected the toxin are two ways people can be exposed to aphantoxin. Animal deaths and human illnesses related to cyanobacterial blooms have been linked to aphantoxin in multiple instances [192]. For aphantoxin in shellfish, the World Health Organization (WHO) has established a provisional guideline value of 20 µg/kg [192,193].

### 4.9. Debromoaplysiatoxin and Aplysiatoxin

**Figure 4:**
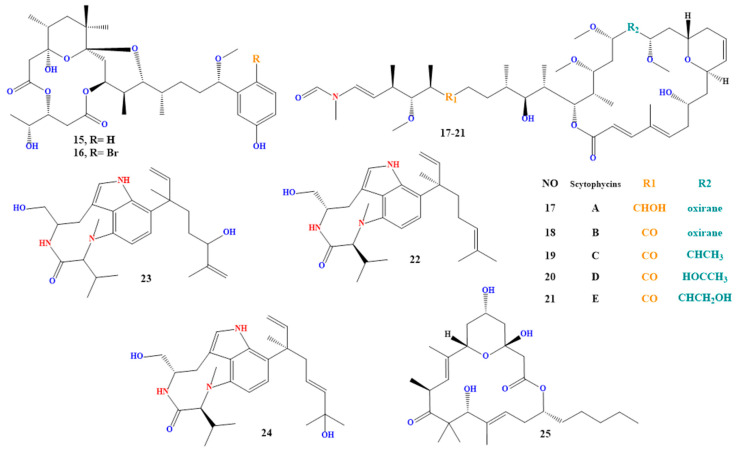
Chemical structures of aphantoxin (**15**), debromoaplysiatoxin (**16**), scytophycins A-E (**17**–**21**), lyngbyatoxins A-C (**22**–**24**), and acutiphycin (**25**).

Aplysiatoxin and debromoaplysiatoxin (compounds **15** and **16** in Figure 4) are lipophilic toxins made by the fungi *Lyngbya majuscula*, *Lyngbya sordida*, and *Schizothrix* [194,195,196,197]. 

It is well known that these poisons are highly poisonous and can have various adverse health impacts on people and animals [190]. Exposure to these poisons can result in liver damage, neurotoxicity, and even death in extreme situations. Additionally, it has been discovered that they possess mutagenic and carcinogenic qualities, raising the risk of cancer. These poisons can be consumed through contact with contaminated water while engaging in recreational activities [196,197,198,199].

### 4.10. Scytophycins and Lyngbyatoxin

Both groups of toxins are lipophilic. *Scytonema hofmanni* produces scytophycins A to E (compounds **17** to **21** in Figure 4), which have been discovered in freshwater and marine settings and are harmful to aquatic and terrestrial species [200,201,202]. While *Moorea producens* and *Lyngbya majuscula* produce lyngbyatoxins A to C (compounds **22** to **24** in Figure 4). It has been demonstrated to have cytotoxic and apoptotic effects on cells [203,204] and is a potent protein synthesis inhibitor.

Exposure to scytophycin and lyngbyatoxin can have a variety of negative health effects on both humans and animals. It has been linked to skin irritation, respiratory problems, and liver damage [205]. Lyngbyatoxin has been demonstrated in lab studies to have a tumor-promoting effect, increasing the risk of cancer. Scytophycin has also been shown to have cytotoxic and apoptotic effects on cells, as well as neurotoxic effects on animals [205,206,207].

### 4.11. Acutiphycin

Acutiphycin is a class of indole alkaloid poisons produced by the genera *Westiellopsis*, *Fischerella*, and *Hapalosiphon* [208,209,210].

Acutiphycin exposure had a variety of adverse health impacts on both people and animals. This toxin’s ability to influence ribosomes and prevent the formation of peptide bonds makes it a potent protein synthesis inhibitor. Animals were also exposed to hepatotoxicity and neurotoxicity [211]. 

Consumption of tainted food sources, such as shellfish and fish, or water can result in ingesting acutiphycin toxin. Additionally, it can be ingested in recreational activities in contaminated water bodies and exposed to aerosolized toxins [209,210,211,212].

## 5. Managing and Mitigating Cyanobacterial Blooms and Toxins

Cyanobacteria can survive and grow in practically any environment. Increased human activity has caused climatic conditions to change, which favors the occurrence and severity of dangerous cyanobacterial blooms everywhere in the world. Even while some environmental variables, such as water temperature, pH, and nutrient levels, may be associated with higher cyanotoxin levels, the interaction of these variables with the particular cyanobacteria species present makes the issue complex. Effective management of a poisonous cyanobacterial bloom requires an understanding of how different environmental conditions affect the regulation of cyanotoxins [213,214,215].

As cyanobacterial blooms can occur in any body of fresh or salt water, it is crucial to know how to spot them. Early detection is essential because it enables quicker response times and more efficient management [216,217]. Additionally, it is critical to comprehend the hazards and probable health issues connected to cyanobacterial blooms [218,219].

**Figure 5:**
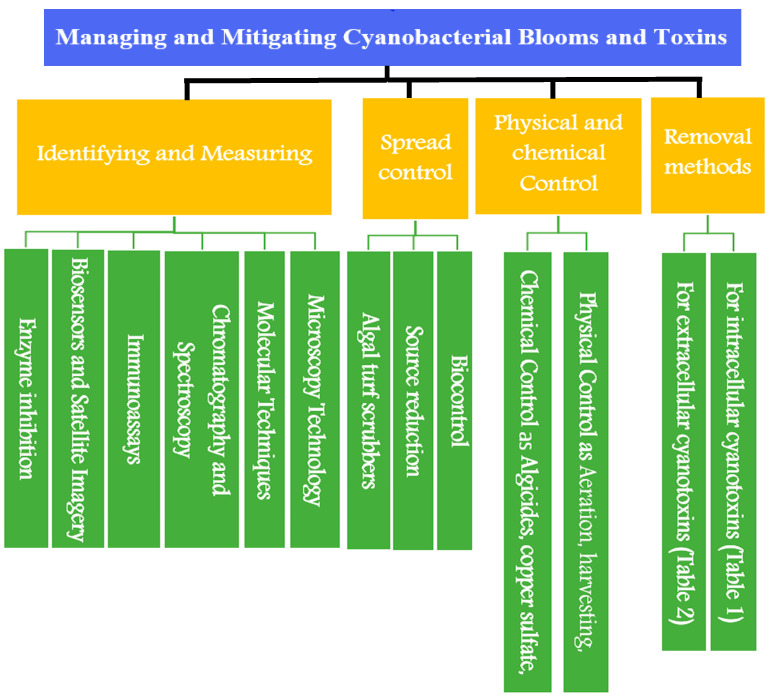
Techniques and methods for managing and mitigating cyanobacterial blooms and toxins.

Monitoring cyanobacteria is a crucial procedure that aids in assessing the condition of water bodies and identifying potential health risks to people and animals. Accurate sample collection is essential for producing results representative of the population. Choosing sampling sites that are indicative of the region of interest, such as those that are close to the coast, in the middle of the water body, or in areas where there is a high likelihood of finding cyanobacteria, are some factors that must be considered while collecting cyanobacteria samples. The site’s accessibility and security must also be considered [220,221,222,223]. Additionally, using sterile and non-toxic sampling tools like a plastic bucket or a sampler made specifically for cyanobacteria sampling. Avoid using metal containers, as they may change the material’s chemical. To obtain a representative sample of the water body, sample the water column vertically as another sampling technique. To capture any fluctuation, take repeated samples at various depths, particularly deep lakes or reservoirs. Each sample should include a minimum of 500 cc of water. Since samples are taken during the cyanobacteria’s active growing season, typically from late spring to early fall, it is crucial to pick the proper time to collect them. To prevent any time-of-day variations, collect samples simultaneously every day. To keep the samples from oxidizing, sodium thiosulfate at 0.1% is added. Within 24 h, transfer samples to the lab while keeping them on ice [223,224,225,226]. Techniques and methods for managing and mitigating cyanobacterial blooms and toxins are summarized in Figure 5.

### 5.1. Identifying and Measuring Cyanobacteria and Cyanotoxins

Cyanobacterial blooms may be identified with the naked eye due to their unusual appearance and color. If a blue-green algal bloom is observed, experts recommend sampling a nearby water source to learn more about the local flora and fauna. Furthermore, scientific examination of bloom samples might reveal the existence of specific bacterial strains and any possible toxins [227,228,229].

Microcystin, saxitoxin, cylindrospermopsin, anatoxin-A, sumoyltoxin A, and lyngbyatoxin A are the most prevalent cyanotoxins that must be watched. Toxin concentrations should be measured as part of a comprehensive risk assessment for aquatic or human health [230,231]. To track the growth of cyanobacteria in lakes and reservoirs, France established the Cyanobacteria Monitoring Network (CyMNet). More than 50 stations comprise this network, tracking variables like temperature, nutritional concentrations, and microcystin toxin levels [232,233,234].

These metrics offer crucial data for tracking and evaluating the dangers posed by cyanobacteria in various global ecosystems. To prevent and mitigate potentially deadly cyanobacterial blooms, one of the most important measures is accurately detecting and measuring cyanotoxins [235,236].

#### 5.1.1. Microscopy Technology 

Cyanobacteria cells are frequently found and recognized using a microscope. A standard method for locating and identifying cyanobacteria cells is light microscopy. The sample is viewed under a microscope when a light source and the cells illuminate. Cyanobacteria cells may be recognized by their distinctive blue-green hue, and the shape and size of the cells can determine different species. Another method for locating and identifying cyanobacteria cells is fluorescence microscopy. In this method, the cells are marked with a fluorescent dye and viewed using a fluorescence microscope. The fluorescent label can distinguish between various cyanobacteria cell types and find toxin concentrations [237,238].

A high-resolution method that can be utilized to see cyanobacteria cells and their structures at the nanoscale level is electron microscopy. In this method, the material is illuminated by an electron beam, and the cells are viewed via an electron microscope. Electron microscopy can be used to identify many structures, including thylakoids and gas vesicles, and can provide precise information on the internal structure of the cells. Confocal microscopy is a method that can be used to produce three-dimensional, high-resolution images of cyanobacteria cells. In this technique, a laser illuminated the sample, and several photographs are taken using various focus planes. A three-dimensional representation of the cells is then created by combining the photos [239,240].

There are not many case studies that show how to employ microscopy to find cyanobacterial cells. The blooms in Lake Taihu, a huge freshwater lake in China, were found during a survey to identify potentially harmful cyanobacterial blooms. The cyanobacterium *Microcystis aeruginosa* bloom caused a water crisis for millions of people in the vicinity. Cyanobacteria were found and identified using microscopy in the lake water sampled. The results of the microscope showed that the dominant cyanobacterium in the bloom was M. aeruginosa, which produces the potent hepatoxin microcystin [241]. Another study used microscopy to monitor the cyanobacterial populations during a 12-month period in a Brazilian eutrophic reservoir. The cyanobacteria population was dominated by *Microcystis*, *Planktothrix*, and *Anabaena*, according the microscope findings. The cyanobacteria communities fluctuated seasonally, with more diversity and abundance in the warmer months, according to another study finding [242].

The disadvantage of microscopic strategies is their lack of accuracy. The detection of microscopic cells is difficult due to the 200 nm resolution limit of optical microscopy. Erroneous cell estimations and non-homogeneous cell distribution may be brought on by the presence of dense colonies. An erroneous estimation of cell abundance results from the diverse dispersion of cells. However, the aggregation state of some Microcystis cyanobacterial colonies enables species identification based on morphology. Furthermore, lethal species identification and bloom lethality prediction are not possible with microscope examination. The inability to distinguish between toxic and non-toxic cyanobacteria, which prevents the risk assessment of a bloom, is increasingly the fundamental drawback of this technique [243,244].

#### 5.1.2. Molecular Techniques 

Cyanobacteria cells can also frequently be found using molecular methods. These methods entail searching for particular DNA identifiers or genes exclusive to cyanobacteria. Cyanobacteria can be found and identified using the molecular technique known as polymerase chain reaction (PCR). Specific DNA sequences of the cyanobacteria are amplified via PCR, making it simple to find and classify them. Other molecular methods include next-generation sequencing (NGS), which can sequence the entire genome of the cyanobacteria and compare it to known sequences in databases to identify the specifics, and fluorescent in situ hybridization (FISH), which uses fluorescently labeled DNA probes to bind to specific DNA sequences in the cyanobacteria cells [245,246,247].

In many studies, researchers used quantitative polymerase chain reaction PCR (qPCR) to locate and measure cyanobacteria in water samples from an Australian river. This is an illustration of how cyanobacterial cells can be found using molecular methods. The researchers extracted DNA from the water samples and used qPCR to amplify and quantify the mcyA gene, which is involved in producing microcystin, a characteristic cyanobacteria toxin. They found that cyanobacteria that produce microcystin were heavily concentrated in the river [248]. 

In a different investigation, quantitative polymerase chain reaction (qPCR) was used to count the cyanobacteria in a Japanese eutrophic lake. The qPCR method, which concentrated on the cyanobacteria’s 16S rRNA gene, facilitate the detection and quantification of a number of species, including *Microcystis*, *Anabaena*, and *Aphanizomenon*. The most common cyanobacterium in the lake, according to the study, was *Microcystis*, and its abundance was positively correlated with both the total nitrogen concentration and water temperature [249]. Additionally, qPCR and fluorescence in situ hybridization (FISH) were used in a study in a reservoir in Spain to identify and quantify harmful cyanobacterial species. FISH was used to determine the cyanobacteria’s physical features, and qPCR experiments concentrated on genes specific to *Microcystis* and *Anabaena*. *Microcystis* was found to be the dominant cyanobacterium in the reservoir and to be capable of producing the hepatotoxin microcystin [250].

qPCR has some limitations, one of which is the decrease in amplification efficiency with the length of the reaction product, despite the significant advantages in the detection and quantification of cyanobacteria and the genes that encode toxins, as well as the ability to detect toxic cyanobacteria in water long before the manifestation of cyanobacterial blooms. Furthermore, the transcription and translation of the toxin’s product by cyanobacteria are not always associated with the presence of an enlarged toxin-encoding gene. As a result, other techniques, such as chemical and biological processes, must be used to integrate and confirm qPCR results [251,252]. 

#### 5.1.3. Chromatography and Spectroscopy

Chromatography and spectroscopy, two trusted techniques for examining in-water cyanobacterial cells, can be used to identify the presence of cyanobacterial pigments. While spectroscopy involves examining molecules via light absorption or reflection, chromatography is based on differences in the rate at which components migrate down a column or thin layer of adsorbent material. These two approaches work together to give researchers a thorough grasp of the toxins in a particular water system. In addition to identifying pigments, spectroscopy provides helpful information about the relative abundance or ratio of various pigments, which helps scientists more precisely assess potential risk levels. Numerous watery pigments, including beta-carotene, chlorophylls a and b, diadinoxanthin, and diatoxanthin, can be detected using chromatography. *Microcystins* and *Cylindrospermopsin*, compounds found in drinking water sources, can be seen by spectroscopy at very low quantities (1ppb) [253,254].

In a Lake Taihu, China investigation, HPLC was used to test and identify the microcystins produced by the common cyanobacterium *Microcystis aeruginosa*, which is responsible for harmful algal blooms. The investigation revealed that *M. aeruginosa* produced several microcystins, including the potent liver toxin microcystin-LR [255]. In another 2018 study—the authors used fluorescence spectroscopy to locate cyanobacteria cells and their toxic byproducts in water samples. Fluorescence spectra were utilized to differentiate between different cyanobacteria cell types and to detect the presence of microcystin in water samples. In order to swiftly and affordably identify cyanobacteria species and their toxins in water samples, fluorescence spectroscopy was used [256]. A portable, high-resolution fiber-optic Raman system and an ab-based Raman system, used for cell detection and identification, were used to study the cyanobacteria diffusion in the Adriatic Sea. In order to distinguish between distinct cyanobacterial species based on their spectral signatures, Raman spectroscopy was discovered to offer great sensitivity and specificity for detecting small amounts of cyanobacterial cells [257].

In a study conducted in a freshwater lake in France using GC/MS, volatile organic compounds (VOCs) produced by the common filamentous cyanobacterial species Anabaena flosaquae were investigated. Depending on the growth stage and environmental variables, A. flosaquae produces a variety of VOCs, including alkanes, alkenes, ketones, and alcohols [258].

Due to the existence of additional substances with comparable absorbance spectra, identification only based on UV absorbance is insufficient. Cyanotoxins are not specifically detected by the diode-array detector, which is also susceptible to interference from other analytes. Results are typically presented as microcystin-LR equivalents because many microcystin variants are difficult to identify and lack standards. Additionally, because some chemicals quickly degrade into anatoxin-A, chromatographic methods combined with UV spectroscopic detection are insufficiently sensitive, necessitating derivatization to increase their detection limits. Better sensitivity and selectivity are made possible by coupling with MS. On the other hand, the various LC-MS techniques make it rather simple and easy to analyze microcystins, nodularin, and cylindrospermopsin. However, utilizing LC-MS techniques to analyze highly polar chemicals with multiple isomers, such as saxitoxins, is extremely challenging [259,260,261].

Due to its outstanding sensitivity and selectivity, liquid chromatography with triple quadrupole mass spectrometry (LC-MS/MS) enables the precise identification and quantification of various and unidentified toxins in environmental materials. These MS/MS transitions are found when specific daugh-terons are produced from the precursor molecular ion in a collision cell [262]. 

m/z 135 [263] is a distinctive fragment ion shared by the majority of microcystins and nodularin. Despite having a different chemical structure from microcystins and nodularins, cylindrospermopsin is frequently analyzed alongside these cyanotoxins via LC-MS/MS using the particular MS-MS transitions m/z 416 > 336, 274, and 194 [264,265]. Anatoxin-a can be detected using the particular MS/MS transitions m/z 166 > 149, 131, 107, and 91 [266,267]. With the precise MS/MS transitions m/z 300 > 282, 204, saxitoxins can be found [268]. Table 1 lists various LC-MS and LC-MS/MS techniques for analyzing various cyanotoxins.

Chromatography techniques have some limits when it comes to detecting cyanotoxins, despite being effective and frequently used for the detection and analysis of many substances, including cyanotoxins. One type of sample complexity is as follows: the isolation and identification of cyanotoxins can be hampered by the presence of a complex matrix of organic and inorganic substances in water samples taken from natural bodies like lakes, rivers, and reservoirs. Reduced sensitivity and accuracy in quantification can be caused by matrix effects [269].

The use of chromatography techniques has drawbacks and limits due to sample complexity and sample preparation. A complex matrix of organic and inorganic chemicals, with varying chemical and physical properties, can obstruct the isolation and detection of cyanotoxins in water samples taken from lakes, rivers, and reservoirs. The sensitivity and accuracy of quantification can be decreased by matrix effects. Detection limits are also considered as one of the limitations of using chromatography techniques, as some cyanotoxins can be present in water at very low concentrations, which requires highly sensitive detection methods. Achieving lower detection limits using chromatography may be limited by factors such as sensitivity of the equipment and interference from the sample matrix. In addition to the development and validation of a chromatographic method for the analysis of cyanotoxins can be time consuming and resource intensive. The need to validate different methods for cyanotoxins and sample matrices can complicate the process [270].

In order to overcome these restrictions, chromatographic techniques are frequently combined with other techniques like immunoassays and biosensors.

#### 5.1.4. Immunoassays 

Immunoassays are frequently employed methods for identifying and measuring cyanotoxins. These methods, which rely on antibodies that are particular to the target cyanotoxin, can offer an efficient way to identify and measure these toxins in various environmental samples [271]. Cyanotoxins can be found using several different immunoassays, such as the following:

##### Enzyme-Linked Immunosorbent Assay (ELISA)

The Enzyme-Linked Immunosorbent Assay (ELISA) is an antibody-based assay which can be either monoclonal, specific for a certain molecule variant, or polyclonal, recognizing several types of molecules. ELISA are rapid and sensitive, and a low level of expertise is required. They are useful as primary quantitative screening tools with limits of detection around 0.1 μg/L, but they are susceptible to interferences that limit their use for quantitative analyses.

Anatoxin, microcystins, and cylindrospermopsin are a few examples of particular cyanotoxins that can be detected and measured using the widely used immunoassay known as ELISA. This method involves adding a sample to a microtiter plate coated with antibodies specific to the target cyanotoxin. A secondary antibody that is connected to an enzyme is introduced after the cyanotoxin in the sample binds to the antibodies. A reaction that the enzyme then catalyzes results in a measurable signal that may be measured using a spectrophotometer [272]. In a study published in 2019, ELISA was used to find anatoxin-a in surface water samples gathered from all around Victoria, Australia, where a cyanobacteria bloom occurred. They found anatoxin-a gene sequences in *Cuspidothrix issatschenkoi*, *Aphanizomenon* sp., *D. circinale*, *Anabaena* sp., and *Oscillatoria* sp., as well as their existence and distribution. The results indicate that the ELISA approach could be used to check water bodies for harmful algal blooms regularly and was successful in detecting anatoxin-a in water samples [272]. Table 1 summarizes the ELISA methods for the detection of microcystins, nodularins, and saxitoxins.

The use of widely accessible Enzyme-Linked Immunosorbent Assay (ELISA) test kits, which do not require pricey equipment or intensive training to use, is one of the most used cyanotoxin testing approaches. Semi-quantitative field screening ELISA kits for detecting the presence or absence of cyanotoxins are available. A repeat study using a quantitative ELISA test or one of the other analytical techniques is recommended if cyano-toxins are found by a field screening kit. More accurate, quantitative ELISA test kits are available for microcystins, nodularins, saxitoxin, anatoxin-a, and cylindrospermopsin. While ELISA kits offer quick results, their selectivity is limited, and they are not congener-specific. Additionally, the quantitative ability of ELISA to distinguish unique variants or congeners of cyanotoxins may vary due to varying cross-reactivities [272,273].

Abraxis Microcystins Kits, Microcystin-ADDA Test Strips, and the Microcystins-DM ELISA Kit are the top three microcystin test kits available today. Microcystin-LR and microcystin-RR-specific antibodies are used in the Abraxis kit’s ELISA to detect microcystins. The strips identify the ADDA moiety present in all types of microcystin. Although they are good for on-site screening, they are not as accurate as the Abraxis ELISA kit. Microcystins-DM from Abraxis employs the ELISA technique as well, but it uses an antibody to detect microcystins by binding to the D-glutamic acid (DM) component of the microcystin molecule. It gives reliable results for the most prevalent microcystin variants but may not pick up on other less prevalent forms [274,275].

##### Protein Phosphatase Inhibition Assay (PPIA)

Microcystins and nodularins, which are potent inhibitors of protein phosphatase, can be detected using a biochemical Protein Phosphatase Inhibition Assay (PPIA). This assay is a simple, rapid, sensitive, and reproducible colorimetric method [268]. The inhibition of eukaryotic protein phosphatases is a well-established indicator of toxin concentration. There are two methods for measuring this inhibition: radio-isotopic techniques using radiolabeled substrates and colorimetric assays using substrates like p-nitrophenyl phosphate [267,273]. The radio-isotopic method relies on radiolabeled proteins and is not suitable for routine monitoring. In the PPIA, the enzyme is exposed to a sample containing the toxin before incubation with the substrate [276]. By measuring the absorbance of the mixture at a specific wavelength, the substrate (or its transformation product) can be detected, and the enzyme activity can be assessed. The enzyme activity is inversely proportional to the concentration of the toxin. The PPIA method facilitates toxin detection within a few hours and can quantify microcystin-LR with a detection limit of 0.01 μg/L [268]. 

However, it is important to note that PPIA cannot differentiate between co-occurring variants of microcystins or distinguish microcystins from nodularins. Therefore, the results are often reported as equivalent microcystin-LR/L. Additionally, when analyzing water containing blooms, potential interferences with unknown compounds may lead to overestimation or underestimation of toxin concentration. Furthermore, since PPIA only detects microcystins and nodularins, further analysis is necessary to detect other cyanotoxins that may be present in the sample [277]. Depending on the class of microcystins, the PPIA method exhibits varying sensitivities to different classes of toxins. It provides a measure of relative toxicity, but it cannot identify the specific toxins [267,268,271,277,278]. A commercially available PPIA test in a 96-well microplate format is currently available [279]. Table 1 shows the analytical PPIA methods for the determination of microcystins and Nodularin. The analytical PPIA methods for determining microcystins and nodularin are displayed in Table 1.

**Table 1:** LC-MS, LC-MS/MS, ELISA, and PPIA methods for the analysis of cyanotoxins in environmental samples.

ID Method	Cyanotoxin	Detection Limit	Sample Preparation	Water Type
LC-MS	Anatoxin-a [280]	0.0021 μg/L	Solid phase extraction disk	Freshwaters
	Microcystins [261]	1 μg/g	Solid phase dispersion	Cyanobacteria strain-cultures water
LCMS/MS	Microcystins [281]	0.002 μg/L	Solid phase extraction	Spiked surfacewater
	Nodularin [282]	0.1 μg/L	Solid phase extraction	Lake water
	Anatoxin-a [283]	0.5 μg/L	Freezing and thawing of cells	Surface waters
	Cylindrospermopsin [284]	0.3 μg/L	Solid phase dispersion	Lake waters
	Microcystins [285]	<0.02 μg/L	Solid phase dispersion	Surface and Drinking waters
ELISA	Saxitoxin [260]	0.02 μg/L	Filtration and sonication	Freshwater ecosystems
	Microcystins [286]	0.05 μg/L	Solid phase dispersion	River
	Nodularin [287]	0.1 μg/L	Filtration	Surface waters
	Microcystin LR in clams [288]	0.1 ng/mL	Solid phase dispersion	Coastal ponds
	Microcystins in mussels [289]	0.1 μg	Lyophilisation	Estuary
PPIA	Microcystins [290]	0.2 μg/L	Freeze drying	Lake water
	Nodularin [291]	Not given	Solid phase dispersion	Lake water
	Microcystins [292]	0.01 μg/L	Filtration	Water supply

#### 5.1.5. Biosensors and Satellite Imagery

Using a biosensor is the most reliable method for determining whether cyanobacteria and their toxins are in the environment. Specialized devices called biosensors use receptors that bind to specific toxins and send out signals when they are present. These sensors can detect low-concentration contaminants in water samples, enabling early detection and prompt action [293]. 

##### Whole-Cell Biosensors

Whole-cell biosensors use entire, living cells as sensing components to find the target analytes’ existence. These biosensors can react to particular cyanobacterial metabolic pathways or molecular markers. By detecting the metabolic activity of the cells and producing an electrical signal, for instance, a biosensor based on immobilized whole-cell cyanobacteria was utilized to determine the presence of cyanobacteria in water samples [293,294].

##### Antibody-Based Biosensors

Immobilized antibodies are used in antibody-based biosensors to selectively capture and detect particular chemicals or biomolecules linked to cyanobacteria. For instance, phycocyanin was found in water samples using a biosensor based on immobilized antibodies. The biosensor measured the binding of phycocyanin to the immobilized antibodies using a fluorescence-based method, which was detected through variations in the fluorescence signal [295].

##### Aptamer-Based Biosensors

To specifically bind and detect particular chemicals or biomolecules connected to cyanobacteria, aptamer-based biosensors use synthetic nucleic acid molecules known as aptamers. A biosensor based on immobilized aptamers was employed to find microcystin in water samples. The biosensor measured the binding of microcystin to the immobilized aptamers using a colorimetric assay, which was detected by variations in the color of the test solution [296].

##### Enzyme-Based Biosensors

Immobilized enzymes are used in enzyme-based biosensors to catalyze particular processes connected to the presence of cyanobacteria cells or their metabolic activity. It can be assumed that a phosphorus-containing substance-based biosensor based on immobilized alkaline phosphatase was employed to identify the molecule’s presence in water samples after cyanobacteria cells discharged it. The biosensor used a colorimetric test to quantify the immobilized enzyme’s activity, which was picked up via alterations in the assay solution’s color [297].

Using satellite imagery as a detection method, authorities can cheaply and effectively monitor broad regions of water thanks to the ability to detect cyanobacteria blooms from space based on changes in the color of the water. To monitor blooms in the future, it is beneficial to pinpoint regions where they have already occurred. The National Oceanic and Atmospheric Administration (NOAA) and the European Space Agency’s Sentinel-3 satellite use satellite photography for cyanobacteria detection and monitoring [298]. 

The Ocean and Land Color Instrument (OLCI) on the Sentinel-3 satellite can identify the spectral signature of cyanobacteria pigments in water bodies. Scientists may recognize and monitor cyanobacteria blooms’ spatial extent and temporal dynamics by examining the OLCI data. The Baltic Sea experienced a significant bloom of poisonous cyanobacteria in 2019 that was observed by the Sentinel-3 satellite and connected to multiple incidences of respiratory sickness in humans and animals [299]. 

The Cyanobacteria Monitoring Program of NOAA uses satellite imagery and other data sources to monitor cyanobacteria blooms throughout the United States. The initiative frequently informs resource managers and the general public about the location and severity of cyanobacterial blooms. Numerous beaches and fishing sites had to be closed as a result of a massive cyanobacteria bloom that occurred in the Florida lake Okeechobee in 2018 [300].

#### 5.1.6. Enzyme Inhibition Methods

To find cyanotoxins in the environment, biochemical approaches are frequently used. Enzyme inhibition techniques are one such highly effective strategy for achieving this. By exposing the material to enzymes, this technique determines how much the enzymes are inhibited. Even in complicated water samples, modest quantities of cyanobacterial toxins can be found using enzyme inhibition techniques. This implies it is an excellent instrument for identifying tiny levels of poisons that other techniques might miss. Additionally, it can detect various poisons, including cylindrospermopsin, anatoxins, and microcystins [301,302]. 

Since cyanobacteria depend on photosynthesis to generate energy, their existence can be detected using photosynthesis inhibitors. Cyanobacteria are found in water samples using a substance known as 3-(3,4-dichlorophenyl)-1,1-dimethylurea (DCMU), a photosynthesis inhibitor. The DCMU stopped the cyanobacteria from producing oxygen, which reduced oxygen production—a cyanobacteria indicator—and was used to ascertain the presence of the cyanobacteria [303]. Others were successful in controlling the growth of bloom-forming M. aeruginosa by employing 2-Hydroxychalcone as a cyanobacterial photosynthesis inhibitor [304]. 

Since cyanobacteria are known to fix nitrogen, their presence can be determined using inhibitors of nitrogen fixation. The acetylene nitrogen fixation inhibitor can be used to find cyanobacteria in sediments from eutrophic lakes. Because the acetylene reduced the cyanobacteria’s ability to fix nitrogen, less ethylene, a cyanobacterium indicator, was produced [305]. Researchers also used L-phenylalanine, a phosphatase inhibitor, to find cyanobacteria in water samples. Because L-phenylalanine inhibits cyanobacterial phosphatase activity, orthophosphate, a cyanobacteria indicator, was secreted less frequently [306].

### 5.2. Control of Cyanobacteria and Cyanotoxins

#### 5.2.1. Strategies for Managing the Spread and Control of Cyanobacteria

Fortunately, many tactics may be used to manage the cyanobacteria’s spread and containment.

##### Biocontrol

One typical biocontrol technique is the introduction of a naturally occurring bacterium that has antagonistic effects on cyanobacteria, either through competition for resources or via the creation of chemicals toxic to cyanobacteria, such as *Bacillus* spp. and *Pseudomonas* spp. [307,308,309]. On the other side, cyanophages, a natural virus that feeds on cyanobacteria cells and infects them, can kill cyanobacteria when introduced into infected waters [310]. Other biocontrol strategies involve adding plant extracts or certain aquatic organisms to waters that are already infested [311,312,313]. Several plant extracts, including *Salvinia molesta* [313,314,315] inhibit cyanobacteria growth. Phytoplanktivorous fishes, including silver carp, big-head carp (*Hypophthalmichthys nobilis*, previously *Aristichys nobilis*) and tilapia (*Oreochromis niloticus*) are direct consumers of phytoplankton (including MC-producing *M. aeruginosa*) and zooplankton, and thus are widely used in the non-traditional bio-manipulation of cyanobacterial blooms [142]. Aquatic creatures that consume cyanobacteria, like filter-feeding invertebrates and zooplankton Daphnia, can aid in lowering their population. Long-term monitoring of this approach can be challenging and it is not always effective [142,315].

##### Source Reduction 

It is a management strategy that aims to reduce the nutrient inputs that support cyanobacteria growth and, as a result, aids in controlling the spread of cyanobacterial blooms. Reducing fertilizer use (primarily nitrogen and phosphorus) on surrounding agricultural fields, reducing wastewater inputs, and regulating storm water runoff are all examples of nutrient management strategies [316,317]. Additionally, vegetation is crucial in controlling cyanobacterial reproduction because it lowers nutrient inputs and offers shade, reducing the quantity of light available for cyanobacterial growth. The restoration or creation of wetlands, the planting of buffer strips, and the application of riparian zone management techniques are among the vegetation management options used there [318,319].

Septic systems are an essential source of nutrients added to water bodies, particularly in places where residences are built close to bodies of water. Nutrient inputs are reduced and the spread of cyanobacteria is controlled through proper septic system management and maintenance. Additionally, soil degradation increases the amount of nutrients added to water bodies, which might promote the growth of cyanobacteria. Soil erosion and nutrient inputs are reduced by implementing soil conservation practices, such as no-till farming and cover crops [315,318,319].

##### Algal Turf Scrubbers (ATS)

By eliminating extra nutrients from bodies of water, algal turf scrubbers (ATS) are a sort of device that can be used to control the growth of cyanobacteria blooms [320,321]. ATS is an algae-growing substrate that is a flow-through channel lined with mesh or another material. Pumping contaminated water through the channel causes the algae to absorb nutrients like nitrogen and phosphorus from the water as it passes over the substrate [322]. After being cleaned up, the water is released back into the environment, which can help control the growth of cyanobacterial blooms [322,323]. ATS can offer additional advantages besides nutrient removal, including carbon sequestration, habitat building, and erosion prevention [323,324,325].

#### 5.2.2. Physical and Chemical Control and Removal of Cyanobacterial Blooms

Physical management and chemical removal techniques can be used to control and reduce cyanobacterial blooms. Chemical removal entails utilizing chemical agents to lower the population of the bacteria or the toxins they produce, whereas physical control involves adding mechanical mechanisms to disturb the blooms [326,327].

##### Physical Control

Cyanobacteria blooms can be physically removed or prevented from spreading using physical control methods. For example, adding oxygen to the water (aeration) can help to disrupt cyanobacteria blooms by encouraging the growth of beneficial bacteria that compete with cyanobacteria for nutrients [328,329]. The water column can be mixed up by aeration, which might lessen the amount of light accessible for cyanobacteria growth. A different approach is to stop their spread by harvesting cyanobacteria from water bodies using nets, screens, or other collecting tools [329,330]. Cyanobacterial blooms can be stopped from spreading to other parts of a body of water by using physical barriers like curtains or booms. In regions where cyanobacteria blooms are concentrated, such as close to a point source of nitrogen inputs, barriers can be beneficial [329,330,331].

By lowering the amount of water available for cyanobacteria growth, water drawdowns can occasionally be employed to control cyanobacteria blooms. This strategy may work well in smaller bodies of water, but it might not be practical or efficient in bigger ones [332,333].

##### Chemical Control

Algicides, copper sulfate, and hydrogen peroxide are a few chemical tools that can be employed to control the growth of cyanobacteria blooms. However, due to potential dangers to the environment and public health, chemical management measures are often only used as a last option [334,335,336,337]. Due to its toxic qualities, copper sulfate mainly kills smaller organisms like cyanobacteria. Still, hydrogen peroxide is frequently employed as a non-toxic substitute for larger organisms or in areas with sensitive species [338,339,340]. Additionally, sodium carbonate peroxyhydrate can be utilized to restrict cyanobacterial growth by removing their ability to photosynthesize by releasing oxygen into the water [339,341]. 

All of these compounds are efficient at lowering cyanobacterial blooms and toxicities. Still, they should only be used when necessary because they might negatively affect the environment in the long term [337,338,339,340,341].

#### 5.2.3. Cyanobacteria Removal Methods

Cyanobacterial cells and small amounts of toxins were successfully eliminated using conventional portable water treatment techniques such as coagulation, sedimentation, filtration, and chlorination [342,343]. The removal strategy, however, must be carefully chosen during a strong bloom when there are significant amounts of cyanobacterial cells and/or their cyanotoxins in the water because some cyanotoxins can be treated with this strategy, while others cannot [343]. 

To choose the best course of action for treatment, one must have a thorough understanding of the cyanobacteria species, growth patterns that make up the majority of the bloom, and the characteristics of the cyanotoxins. Cyanotoxins that are intracellular and extracellular (dissolved) can be removed using various techniques. Numerous techniques have been utilized to remove internal cyanotoxins, including coagulation/sedimentation, coagulation-dissolved air flotation (DAF), micro- and ultrafiltration, and pre-treatment oxidation [343,344,345,346,347,348,349]. While some techniques for eliminating extracellular (dissolved) cyanotoxins have been mentioned in the literature, these techniques include membranes (nano- and ultrafiltration), potassium permanganate, ozonation, the use of free chlorine, ultraviolet radiation, physical cyanotoxin adsorption using powdered activated carbon (PAC), granular activated carbon (GAC), and activated carbon adsorption [350,351,352,353,354]. Each method has benefits and is effective, and the efficacy depends on several variables, including chemical, physical, and biological variables [342,349].

Table 2 shows how well various water treatment methods work to get rid of intact cyanobacteria cells, and Table 3 shows how well different treatment methods work to eliminate extracellular dissolved toxins from many of the most important cyanobacteria.

## 6. Summary and Conclusions

The risks linked with cyanobacteria, sometimes referred to as blue-green algae, spreading in bodies of water are discussed in the manuscript. When conditions are ideal, cyanobacteria can spread swiftly. Their growth may be aided by surplus nitrogen and phosphorus from sewage and fertilizer runoff sources. The toxins they produce are toxic to both people and aquatic life.

This review also outlines the characteristics of many cyanobacterial taxa. *Oscillatoria*, *Microcystis*, *Anabaena*, and other organisms are listed. Numerous cyanobacterial species, such as microcystins, nodularin, anatoxin-a, and cylindrospermopsin, can produce toxins that harm the liver and nervous system. 

Overabundances of nutrients, warm temperatures, lots of sunlight, and slow water flow all encourage cyanobacteria blooms. Sewage treatment facilities and industrial and agricultural endeavors provide the nutrients required for cyanobacteria to flourish.

Different techniques are discussed for finding and getting rid of cyanobacteria. Cyanobacteria cells and their toxins can be found using microscopy, molecular methods, chromatography, and spectroscopy. Biosensors and immunoassays are also reliable detection techniques. Large bodies of water can be monitored with satellite images.

The manuscript also discussed ways to control and lessen cyanobacteria problems. These techniques include physical or chemical treatments, fertilizer input reduction, the use of algal lawn scrubbers, and biocontrol utilizing antagonistic bacteria. These solutions strive to reduce the dangers that cyanobacterial blooms and their toxins offer. Cyanobacteria in water systems must be managed effectively, which requires early diagnosis and quick action.

Risks associated with abridgment and cyanobacteria dispersion call for thorough monitoring and mitigation measures. Management of this crucial environmental issue can be enhanced by better understanding the elements that encourage blooms and the many detection and eradication options available.

## Figures and Tables

**Table 2:** A summary of the effectiveness of removal methods for intracellular cyanotoxins.

Treatment Technique	Effectiveness of the Technique
Oxidation (pre-treatment) [343]	Oxidation stresses or kills cyanobacteria cells that release cyanotoxin into the water.
Coagulation/Sedimentation/Filtration [346,347]	After sludge separation, it must be guaranteed that the supernatant sludge does not re-enter the supply.
Filter membranes [349]	Intracellular cyanotoxins (cyanobacteria cells) can be effectively removed. Microfiltration and ultrafiltration work well when cells are not allowed to accumulate on membranes for extended periods of time. More frequent cleaning may be required during a bloom episode.
Flotation [350]	Since many cyanobacteria that produce toxins are buoyant, flotation techniques like Dissolved Air Flotation (DAF) are effective at removing cyanotoxins from the body.

**Table 3:** A summary of the effectiveness of removal methods for extracellular cyanotoxins.

Treatment Technique	Effectiveness of the Technique
Filter membranes [350]	- Cell lysis is highly probable, albeit influenced by the type of membrane material, how the membrane pore sizes are distributed, and the caliber of the influent water. - Nanofiltration is often effective at eliminating extracellular microcystins. - Reverse osmosis filtering is frequently used to eliminate cylindrospermopsin and microcystins.
KMnO_4_ [351]	Microcystins and anatoxins are effectively oxidized, whereas saxitoxin is not.
Ozone [352]	Microcystins, anatoxin-a, and cylindrospermopsin are all quite effective at being oxidized. Saxitoxin oxidation is ineffective.
Free Chlorine [351]	As long as the pH is less than 8, it is effective for oxidizing microcystins. Cylindrospermopsin and saxitoxin are effectively oxidized. Anatoxin-a oxidation is ineffective.
Ultraviolet radiation [354]	Microcystins and cylindrospermopsin cannot be oxidized by UV radiation alone at dosages typically used in drinking water treatment. UV light is effective at oxidizing anatoxin-a, cylindrospermopsin, and, at high UV dosages, microcystins when mixed with ozone or hydrogen peroxide.
Activated Carbon Adsorption [354]	- The efficiency of PAC adsorption varies with carbon type, pore size, cyanotoxin type, and other indicators of water quality such the amount of natural organic matter (NOM). For instance, activated carbons derived from wood are frequently the best in adsorbing microcystins. The efficiency of GAC (granulated activated carbon) adsorption varies with carbon type, pore size, cyanotoxin type, and other aspects of water quality, including NOM level. GAC is effective against microcystins and is predicted to be effective against saxitoxin, anatoxin-a, and cylindrospermopsin.

## Data Availability

Not applicable.
